# The Effects of Laxogenin and 5-Alpha-hydroxy-laxogenin on Myotube Formation and Maturation During Cultured Meat Production

**DOI:** 10.3390/ijms26010345

**Published:** 2025-01-02

**Authors:** Jeong Ho Lim, Syed Sayeed Ahmad, Ye Chan Hwang, Ananda Baral, Sun Jin Hur, Eun Ju Lee, Inho Choi

**Affiliations:** 1 Department of Medical Biotechnology, Yeungnam University, Gyeongsan 38541, Republic of Korea; lim2249@naver.com (J.H.L.); sayeedahmad4@gmail.com (S.S.A.); 1230hyc@naver.com (Y.C.H.); ananbaral@gmail.com (A.B.); gorapadoc0315@hanmail.net (E.J.L.); 2 Research Institute of Cell Culture, Yeungnam University, Gyeongsan 38541, Republic of Korea; 3 Department of Animal Science and Technology, Chung-Ang University, Anseong 17546, Republic of Korea; hursj@cau.ac.kr

**Keywords:** culture meat, MSC differentiation, anti-ROS, MTSN inhibitor, laxogenin

## Abstract

Cultured meat (CM) is derived from the in vitro myogenesis of muscle satellite (stem) cells (MSCs) and offers a promising alternative protein source. However, the development of a cost-effective media formulation that promotes cell growth has yet to be achieved. In this study, laxogenin (LAX) and 5-alpha-hydroxy-laxogenin (5HLAX) were computationally screened against myostatin (MSTN), a negative regulator of muscle mass, because of their antioxidant properties and dual roles as MSTN inhibitors and enhancers of myogenesis regulatory factors. In silico analysis showed LXG and 5HLXG bound to MSTN with binding free energies of −7.90 and −8.50 kcal/mol, respectively. At a concentration of 10 nM, LAX and 5HLAX effectively inhibited the mRNA and protein expressions of MSTN, promoted myogenesis, and enhanced myotube formation and maturation. In addition, by acting as agonists of ROS downregulating factors, they exhibited antioxidative effects. This study shows that supplementation with LAX or 5HLAX at 10 nM in CM production improves texture, quality, and nutritional value. We believe this study fills a research gap on media development for myotube formation and maturation, which are important factors for large-scale in vitro CM production that improve product quality, nutritional value, and efficacy.

## 1. Introduction

Cultured meat (CM) is a lab-grown meat developed by inducing myogenesis of muscle satellite (stem) cells (MSCs) [1,2], and thus, does not involve the slaughter of livestock. The activation and proliferation of MSCs and the fusion of differentiating myoblasts into mature myofibers under the direction of muscle regulatory factors [3,4], growth factors [5,6], and cytokines [7,8] to produce skeletal muscle (SM), is termed myogenesis [9]. SM contains 50–75% of all body proteins [10], and consumed meat is composed of ~90% SM, ~10% connective and fat tissues, and ~1% blood [11]. Currently, CM production is a hot topic in food science and engineering because it offers a promising alternative protein resource [12] and essential nutrients [13]. Meat is composed of myofibrillar, sarcoplasmic, and matrix proteins. Myofibrillar proteins are rich in essential amino acids, which are effectively absorbed by the human body [14], and have excellent water-holding capacities and gelation and emulsification characteristics that contribute to the mouthfeel of meat [15,16]. Therefore, CM should contain a suitable amount of myofibrillar protein.

The availability of proteinaceous foods is certain to become existential over the coming decades because it has been predicted that the world’s population will reach around 9 billion by 2050, and thus, sustainable CM production is a critical goal [17,18]. In addition, it should be added that CM has other advantages over regular meat, including environmental and ethical considerations, water and environmental pollution, and land use [18].

MSCs are precursors of the muscle fibers that form SM via myogenesis [6]. CM was initially made from bovine MSCs [19], and co-culture of MSC and adipocytes contributed to the taste and texture of animal meat [8]. Furthermore, MSC culture and differentiation [20,21] offer innovative means of achieving myogenesis in vitro. Several extracellular matrix (ECM) proteins such as fibromodulin [22,23], matrix gla protein [24], and membrane proteins like IgLON4 [25] and IgLON5 [22] have been reported to regulate myogenesis and provide structural support and cellular communication and contribute to the architectural maintenance of SM [21,26]. In previous studies, we explored the roles of ECM [26], growth factors and hormones [6], cell and myokine types [8], and natural compounds [27] on the efficacy of CM production.

By inhibiting myoblast differentiation, MSTN has a major negative effect on SM formation [28]. MSTN is produced by skeletal myofibers, circulates in blood, and acts on myofibers to limit SM growth [29]. MSTN binds with its receptor ACVR2b (activin receptor type-2B) and thus activates signaling for protein degradation through Smad2/3-mediated transcription [30]. Several MSTN inhibitors [27,31,32,33] and peptides [34], have been reported to increase muscle mass [28], and some other natural small compounds have been reported to increase myogenic differentiation [35]. In addition, some natural compounds have been shown to enhance the self-renewal and differentiation abilities of MSCs [36], the latter of which is accompanied by the assembly of myofibrils and the maturation of myosin and actin [37]. Many challenges associated with CM production have yet to be resolved. In particular, a cost-effective medium that promotes cell growth and differentiation during the production process is probably the most important. In this context, it seems reasonable to identify or screen natural compounds that have already been proven safe for human use for CM production.

Mammalian cells are widely used in the food, pharmaceutical, and medical industries, and developing suitable culture media is essential to achieve the performance required for cell culture engineering [38]. The goal of this study was to develop a cost-effective dietary additive/supplement that inhibits MSTN, and thus, promotes the in vitro formation of a large number of mature myotubes through MSC differentiation for the mass production of CM. In silico screening of 75 natural compounds found in garlic for MSTN activity identified laxogenin (LXG) as a potential MSTN inhibitor. A subsequent analysis of 5HLXG (5-alpha-hydroxy-laxogenin, a derivative of LXG) showed it had a similar effect. LXG is a component of garlic (*Allium sativum*) and several other plants, including *Smilax sieboldii*, *Allium schoenoprasum*, *Allium chinense*, and *Solanum unguiculatum*, and 5HLXG has been associated with muscle mass gain. In the present study, LXG and 5HLXG were subjected to in vitro study to determine whether, as media components, they inhibit MSTN, enhance MSC differentiation, and provide a potential means of producing CM.

## 2. Results

### 2.1. In Silico Analysis

**Figure 1:**
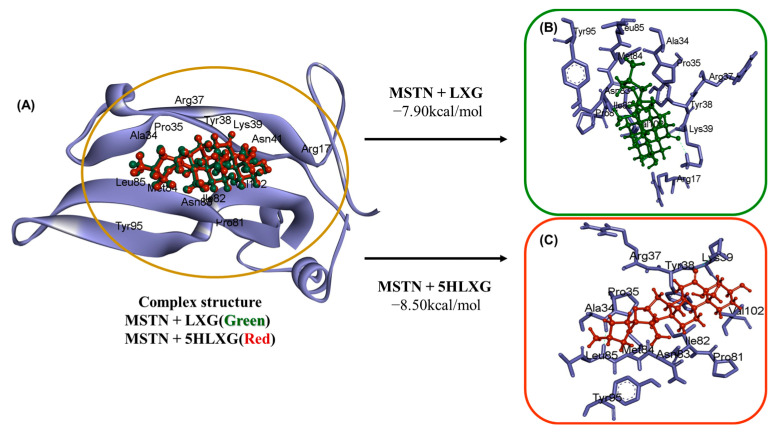
The interactions between LXG and 5HLXG with MSTN. (**A**) The amino acid residues of MSTN that interact with LAX or 5HLAX during complex formation. (**B**) Interaction between MSTN and LAX. (**C**) Interaction between MSTN and 5HLAX. Five hydrophobic interactions were observed during MSTN + LXG and MSTN + 5HLXG complex formation (Table 1).

Analysis showed LXG and 5HLXG bound to the Arg17, Ala34, Pro35, Arg37, Tyr38, Lys39, Asn41, Pro81, Ile82, Asn83, Met84, Leu85, Tyr95, and Val102 amino acid residues of MSTN with free energies of –7.90 and −8.50 kcal/mol, respectively (Figure 1A). The docking study showed two H-bonds, namely, LYS39:HN—LIG0:O3 and LYS39:HZ3—LIG0:O3 in MSTN + LXG complex (Figure 1B), and LYS39:HN—LIG0:O4 and LYS39:HZ3—LIG0:O4 in MSTN + 5HLXG complex were formed by the interactions (Figure 1C).

**Table 1:** Amino acid residues involved in the interaction between MSTN and LXG or 5HLXG.

Parameters	MSTN + LXG	MSTN + 5HLXG
Binding energy (kcal/mol)	−7.90	−8.50
Amino acids involved	Arg17, Ala34, Pro35, Arg37, Tyr38, Lys39, Asn41, Pro81, Ile82, Asn83, Met84, Leu85, Tyr95, Val102	Ala34, Pro35, Arg37, Tyr38, Lys39, Pro81, Ile82, Asn83, Met84, Leu85, Tyr95, Val102
H-bonds	LYS39:HN—LIG0:O3 LYS39:HZ3—LIG0:O3	LYS39:HN—LIG0:O4 LYS39:HZ3—LIG0:O4
Hydrophobic interactions	LIG0—ILE82 LIG0:C31—ALA34 LIG0:C31—LEU85 PRO35—LIG0 VAL102—LIG0	LIG0—ILE82 LIG0:C32—ALA34 LIG0:C32—LEU85 PRO35—LIG0 VAL102—LIG0

In addition, the toxicity and absorption parameters of LXG and 5HLXG were checked. The human intestinal absorptions of LXG and 5HLXG were 97.12 and 96.78%, respectively, and there was no evidence of AMES toxicity, hepatotoxicity, or skin sensitization (Table 2), which suggested that LXG and 5HLXG are highly absorbed by the human intestine and do not have toxic effects.

### 2.2. In Vitro Analysis

#### 2.2.1. Effects of LXG and 5HLXG on the Proliferations of Bovine, Porcine, and Chicken MSCs and C2C12 Cells

MSCs and C2C12 cells proliferations were assessed after treating cells during the first 4 days with LXG or 5HLXG at 0.1, 1, 10, 100, or 1000 nM. At these concentrations, neither LXG nor 5HLXG had a significant effect on cell proliferation as determined by the MTS assay (Supplementary Figures S1–S4)

#### 2.2.2. Effects of LXG and 5HLXG on the Differentiation of Bovine MSCs

**Figure 2:**
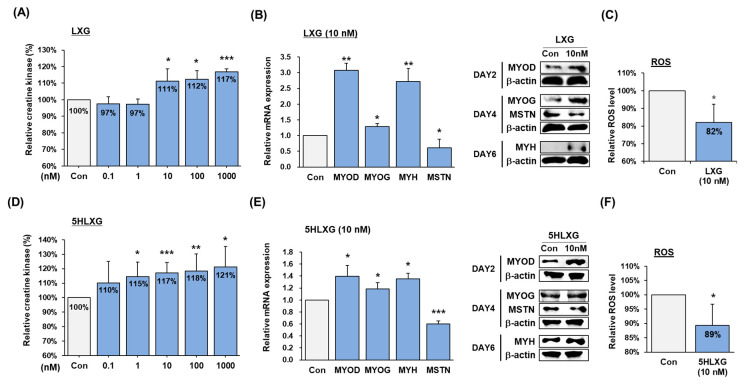
Differentiation of bovine MSCs treated with LXG or 5HLXG. (**A**,**D**) Differentiation was assessed using a creatine kinase activity assay on LXG and 5HLXG treated cells. (**B**,**E**) mRNA and protein levels of myogenic markers and MSTN were determined by Real-time RT-PCR and Western blot. (**C**,**F**) ROS levels in LXG and 5HLAX treated cells were determined using a 2′,7′-dichlorofluorescein assay. Means ± SD (n > 3). * *p* ≤ 0.05, ** *p* ≤ 0.01, *** *p* ≤ 0.001.

Creatine kinase activity increased when LXG or 5HLXG were added to bovine MSC differentiation medium. LXG increased creatine kinase activity by 11% at 10 nM, while 5HLXG increased it by >15% at 1 nM (Figure 2A,D). Myotube width and length in LXG or 5HLXG supplemented differentiation medium were larger than in control medium (Figure S5A,B). At 10 nM both LXG and 5HLXG increased the mRNA and protein expression levels of MYOD (early muscle differentiation marker), MYOG (myogenin, markers for myotube formation), and MYH (myosin heavy chain, maturation markers of myotube), whereas the expression of MTSN, which was expected to bind to LXG, was reduced (Figure 2B,E). Further, at 10 nM LXG and 5HLXG reduced ROS levels by 18% and 11%, respectively (Figure 2C,F).

#### 2.2.3. Effects of LXG and 5HLXG on Porcine MSC Differentiation

**Figure 3:**
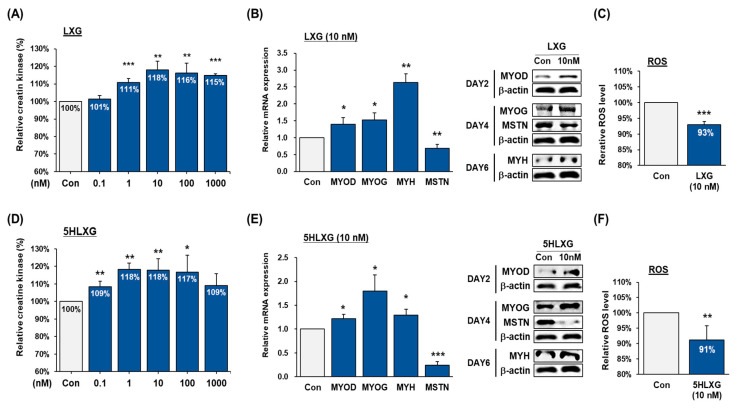
Differentiation of porcine MSCs treated with LXG or 5HLXG. (**A**,**D**) Differentiation was assessed using a creatine kinase activity assay. (**B**,**E**) mRNA and protein levels of myogenic markers and MSTN were determined by Real-time RT-PCR and Western blot. (**C**,**F**) ROS levels were determined using a 2′,7′-dichlorofluorescein assay. Means ± SD (n > 3). * *p* ≤ 0.05, ** *p* ≤ 0.01, *** *p* ≤ 0.001.

The effects of LXG and 5HLXG on the differentiation of porcine MSCs were checked at 0.1, 1, 10, 100, and 1000 nM. LXG increased the activity of muscle-specific creatine kinase by up to 18% at 10 nM, and 5HLXG increased its activity significantly by ~9% at 0.1 nM and 18% at 1 and 10 nM (Figure 3A,D). At 10 nM, both LXG and 5HLXG effectively increased the differentiation of porcine MSCs. In LXG-supplemented media, the mRNA levels of MYOD, MYOG, and MYH increased by approximately 40%, 50%, and 150%, respectively, compared to the control, and corresponding protein expressions were also increased. However, the mRNA and protein levels of MSTN decreased (Figure 3B). 5HLXG also increased MYOD, MYOG, and MYH levels at the mRNA and protein levels in differentiating porcine MSCs and reduced MSTN mRNA and protein expressions (Figure 3E). Furthermore, at 10 nM, LXG and 5HLXG reduced ROS levels by 7% and 9%, respectively (Figure 3C,F).

#### 2.2.4. Effect of LXG and 5HLXG on Chicken MSC Differentiation

**Figure 4:**
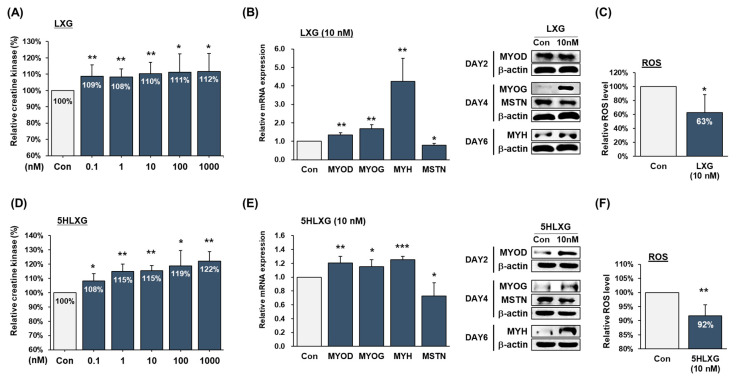
Differentiation of chicken MSCs treated with LXG or 5HLXG. (**A**,**D**) Differentiation was assessed using a creatine kinase activity assay. (**B**,**E**) mRNA and protein levels of myogenic markers and MSTN were determined by real-time RT-PCR and Western blot. (**C**,**F**) ROS levels were determined using a 2′,7′-dichlorofluorescein assay. Means ± SD (n > 3). * *p* ≤ 0.05, ** *p* ≤ 0.01, *** *p* ≤ 0.001.

LXG or 5HLXG supplementation increased chicken differentiation by 9% to 12% and up to 22%, respectively (Figure 4A,D). At 10 nM, LXG significantly increased MYOD and MYOG mRNA levels and particular MYH mRNA levels by more than 4-fold; protein expressions were also increased. Additionally, at 10 nM, 5HLXG increased MYOD, MYOG, and MYH mRNA and protein levels. These results concur with bovine and porcine results. In addition, MSTN mRNA and protein levels decreased after treatment with LXG or 5HLXG during the differentiation of chicken MSCs (Figure 4B,E). Regarding the effects of LXG and 5HLXG on ROS generation during differentiation, 10 nM LXG reduced ROS levels by 37%, and 10 nM 5HLXG caused an 8% reduction versus non-treated controls. These results confirmed that both LXG and 5HLXG can reduce ROS generated during the differentiation of chicken MSCs (Figure 4C,F).

#### 2.2.5. Effects of LXG and 5HLXG on C2C12 Cell Differentiation

**Figure 5:**
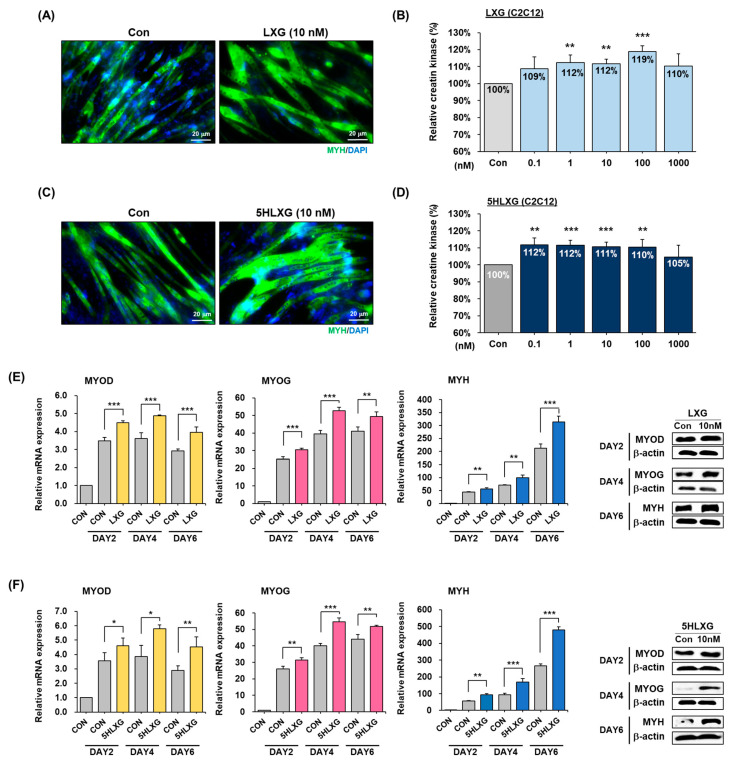
Differentiation of C2C12 cells treated with LXG or 5HLXG. (**A**,**C**) Myotube formation was observed by immunocytochemistry for MYH. (**B**,**D**) Differentiation was assessed using a creatine kinase activity assay. (**E**,**F**) mRNA and protein levels of myogenic markers were determined by Real-time RT-PCR and Western blot. Means ± SD (n > 3). * *p* ≤ 0.05, ** *p* ≤ 0.01, *** *p* ≤ 0.001.

C2C12 cells were treated with LXG or 5HLXG up to differentiation day 4. Myotube lengths and widths in both LXG and 5HLXG-supplemented cells (10 nM) increased versus non-treated cells. In addition, MYH protein expression (green) was observed by immunocytochemistry and found to be elevated in treated cells (Figure 5A,C). In addition, muscle-specific creatine kinase activities were compared after treating cells with LXG and 5HLXG at different concentrations. When LXG was treated at different concentrations during the differentiation of C2C12 cells, creatine kinase activity was increased from 9% to 19% at 0.1 to 100 nM. In the case of 5HLXG, an increase of ~12% was observed at 0.1 nM, and increases were observed at 1, 10, and 100 nM, respectively (Figure 5B,C). The effects of LXG and 5HLXG (10 nM) on the muscle differentiation process at different time points (0, 2, 4, and 6 days) were confirmed using the mRNA and protein expressions of MYOD, MYOG, and MYH as determined by real-time RT-PCR and Western blot. When LXG or 5HLXG were supplemented at 10 nM, early/mid (MYOD) and late muscle marker gene (MYOG and MYH mRNA) expressions and protein levels were both significantly increased (Figure 5E,F).

#### 2.2.6. Inhibition of MSTN and ROS Suppression by LXG or 5HLXG Supplementation

**Figure 6:**
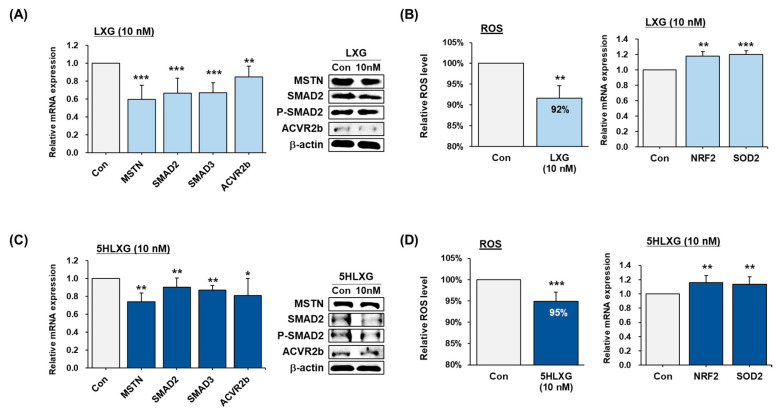
Effects of LXG and 5HLXG on the differentiation of C2C12 cells. (**A**,**C**) mRNA and protein levels of MSTN, SMAD2, SMAD3, and ACVR2b were determined by real-time RT-PCR and Western blot. (**B**,**D**) ROS levels were determined using a 2′,7′-dichlorofluorescein assay and mRNA levels of NRF2 and SOD2. Means ± SD (n > 3). * *p* ≤ 0.05, ** *p* ≤ 0.01, *** *p* ≤ 0.001.

Treatment of C2C12 cells and differentiating bovine, porcine, and chicken MSCs with LXG or 5HLXG decreased MSTN mRNA and protein levels. In addition, treatments suppressed the mRNA levels of SMAD2 and 3 (intracellular MSTN signaling molecules), SMAD2 protein and phosphorylated SMAD2 levels, and the mRNA and protein levels of ACVR2b (the cell membrane receptor of MSTN) (Figure 6A,C). LXG or 5HLXG also significantly reduced ROS levels by ~8% and 5%, respectively. In addition, the mRNA level of NRF2 (a transcription factor known to reduce ROS) and SOD2 (an enzyme that directly removes ROS) increased when cells were treated with LXG or 5HLXG at 10 nM (Figure 6B,D).

## 3. Discussion

Population expansion, increasing animal consumption, and limited food supplies all hint at some future food supply calamity. Accordingly, the need for dietary protein will increase, and as a result, cultured meat is being developed as an alternative to animal-derived products [16,39]. Currently, a lack of systematic research on the texture and protein quality of CM makes it difficult to identify better means of producing CM on a large scale. However, recent studies on the in vitro proliferation and differentiation of SM have extended beyond medical applications to include techniques for boosting livestock output such as CM-producing techniques. CM production is highly dependent on myogenesis, which converts cells into edible meat. Unfortunately, studies in the CM sector on myogenic differentiation process are limited. Given this background, IGF-1 is used in bovine MSC differentiation media to increase myotube formation [40], luteolin is used to activate the PI3K/AkT/mTOR pathway to increase MYH3 [41], and quercetin is used to promote the differentiation of bovine, chicken, and porcine MSCs for CM production [27]. However, there is a huge shortcoming in the amount of research being undertaken on the development and maturation of myofibers for CM production.

In this study, an in silico investigation was used to screen 75 natural compounds against MSTN, a negative regulator of muscle development and growth [31,42]. MSTN and its influence on meat output have been extensively researched. MSTN is highly conserved in mammals, and loss-of-function or mutations can result in increased SM weight and a double-muscle phenotype in several livestock animals, such as cattle, sheep, and pigs, and in other species, including rabbits and humans [43,44,45]. Here, LXG and 5HLXG were identified as potential MSTN inhibitors, and in vitro studies showed that both, when added to media, promoted the differentiation of bovine, chicken, and porcine-derived MSCs. In addition, we repeated the study to determine the effects of LXG and 5HLXG on myotube formation and maturation by C2C12 cells, a well-stabilized myoblast cell line. Our findings suggested that when supplemented in media, LXG and 5HLXG can produce CM, which is consistent with previous studies in which naringenin and quercetin showed similar effects [27,46]. Furthermore, our data show that LXG or 5HLXG have antioxidant properties and lower ROS levels during cell expansion or differentiation. Specifically, at 10 nM, LXG and 5HLXG promoted the differentiation of bovine, porcine, and chicken-derived MSCs and C2C12 cells by significantly increasing MYH expression, thus indicating myotube generation, an important requirement for CM production.

LXG and 5HLXG were found to bind to MSTN with free energies of −7.90 and −8.50 kcal/mol, respectively. The docking study showed the formation of two H-bonds in MSTN + LXG and MSTN + 5HLXG complexes. These two ligands were also checked for their toxicity and absorption parameters. The human intestinal absorptions of LXG and 5HLXG were found to be 97.12 and 96.78%, respectively, and no evidence of AMES toxicity, hepatotoxicity, or skin sensitization was apparent (Table 2), suggesting that these compounds are well absorbed by the human intestine without any toxic effect. Toxicity can empower the discovery of new compounds and computational approaches for toxicity prediction have been established [47,48]. Chemical toxicity assessment for a compound is crucial for health and safety.

MSTN binds with ACVR2b and thus activates signaling for protein degradation through Smad2/3-mediated transcription [30]. Here, LXG, and 5HLXG were found to be inhibitors of MSTN, ACVR2b, SMAD2, and SMAD3 (Figure 6A) during the differentiation of C2C12 cells, which suggests they might be used as media supplements for CM production. It has also been reported that MSTN functions as a pro-oxidant that signals ROS generation in SM and that MSTN treatment significantly increases ROS levels in C2C12 cells [49]. We observed that LXG and 5HLXG inhibited MSTN expression at 10 nM and significantly reduced ROS levels (Figure 6A–D). Additionally, NRF2 (nuclear factor erythroid 2–related factor 2) and superoxide dismutase 2 (SOD2) expressions were significantly increased by LXG and 5HLXG, which would negatively affect ROS levels that are usually considered to be harmful to cells [50]. The effects of reactive oxidants on cells are counterbalanced by complex antioxidant defense systems, and NRF2 is a major contributor to oxidative stress resistance [51]. It has been reported that NRF2 knockout in mice is associated with oxidative pathologies and that NRF2 upregulation protects animals from oxidative damage [52]. In this regard, LXG and 5HLXG at 10 nM significantly increased NRF2 expression. In addition, SOD2 removes mitochondrial ROS and protects against cell death [53]. SOD2 expression was significantly increased by LXG or 5HLXG, further supporting that LXG and 5HLXG powerfully protect cells in cultured media against cell growth and differentiation-induced ROS increases by raising NRF2 and SOD2 levels. Interestingly, recent studies on myogenesis have revealed that ROS effectively controls muscle growth [54,55].

MSTN induces the degradation of myofibrillar proteins [49], such as MYH, an important differentiation and maturation factor required for large-scale CM production, and thus hinders CM production. On the other hand, myofibrillar proteins are required to flavor meat and provide acceptable mouthfeel. LXG or 5HLXG at 10 nM, MYH expression was elevated, and myotube lengths and widths were significantly greater than those of non-treated controls (Figure 5A,C). These observations mean that LXG or 5HLXG could be used for CM production and taste enhancement by increasing myofibrillar protein content.

Research is ongoing to improve the quality of CM, addressing limitations like flavor, texture, meat color, and nutritional content, which differ from original meat. As a promising future technology (CM production), it offers advantages like being less sensitive to climate conditions than traditional meat production. However, economic challenges remain, including issues with cell acquisition, mass production, cost, and the development of suitable media for large scale CM production. These factors, along with production costs and scalability, limit the accessibility and acceptance of CM in the market. Additionally, studies in the CM sector on myogenic differentiation process are limited for large-scale CM production. Furthermore, basic research on mechanisms and influencing factors related to CM production is necessary. Natural compounds as media supplements would be valuable factor for enhancing the MSC proliferation and differentiation for large-scale CM production.

## 4. Materials and Methods

### 4.1. In Silico Investigation

Seventy-five natural compounds found in garlic were identified using different literature sources related to human health management. The structure of these compounds was obtained from the PubChem database (https://pubchem.ncbi.nlm.nih.gov/, accessed on 22 May 2024) and prepared using Discovery Studio (DS) for a docking study. MSTN structure was obtained from the RCSB-PDB (https://www.rcsb.org/, accessed on 22 May 2024) and heteroatoms were removed from MSTN with the help of DS. Autodock [56] was performed at the dimensions of X = −29.37, Y = −20.90, and Z = 21.61 to investigate the affinities of the natural compounds, and the interaction between MSTN and the natural compounds was checked. LXG and 5HLXG showed potential binding affinity to MSTN. The 75 selected compounds were also checked for intestinal absorption and toxicity using pkCSM [57]. Finally, LXG and 5HLXG were forwarded to an in vitro study for MSC proliferation, differentiation, and myotube formation/maturation.

### 4.2. In Vitro Process

#### 4.2.1. MSC Isolation (Bovine, Porcine, and Chicken)

Bovine (17 weeks old) top-round, a male porcine (3 days old), or chicken muscles (16 days after fertilization) were collected. They were crushed and digested by pronase (0.1%) (Roche, Mannheim, Germany) at 37 °C for 1 h, and centrifuged for 3 min (at 1000× *g*). Then, they were filtered with cell strainer (100 µm) (Millipore, Darmstadt, Germany) and suspended in Ham’s F-10 + 20% FBS + 1% P/S + 5 ng/mL FGF2 (fibroblast growth factor 2) medium. Next, they were seeded on collagen-coated plates and placed in a 5% CO2 humidified incubator at 37 °C. MSCs were isolated following the method described earlier [27]. Furthermore, a concentration of 10 nM, which is commonly effective for both LXG and 5HLXG during differentiation, was used. However, based on creatine kinase activity results, the concentration at which effects begin to appear varies slightly across species. We acknowledge this species specificity and are currently investigating the optimal concentration that produces the best effects for each species. Furthermore, we utilize this information to develop customized media supplements optimized for each species.

#### 4.2.2. The Proliferation and Differentiation of MSCs

MSCs were cultured in Ham’s F-10-based growth medium with 0, 0.1, 10, 100, or 1000 nM of LXG or 5HLXG. After 90% confluence, differentiation medium (2% FBS and 1% P/S) was used containing 0, 0.1, 10, 100, or 1000 nM of LXG or 5HLXG. Cells were then incubated for 2, 4, or 6 days at cultured condition.

#### 4.2.3. C2C12 Myoblast Proliferation and Differentiation

C2C12 cells (Korean Cell Line Bank, Seoul, Republic of Korea) were cultured in a DMEM supplemented with 10% FBS and 1% penicillin and containing 0, 0.1, 10, 100, or 1000 nM of LXG or 5HLXG in a humidified 5% CO2 incubator at 37 °C. After 90% confluence, the growth medium was replaced with differentiation medium containing 2% FBS, 1% P/S, and 0, 0.1, 10, 100, or 1000 nM of LXG or 5HLXG. The cells were then cultured for 2, 4, or 6 days.

#### 4.2.4. Ethical Considerations

The guidelines supplied by the Institutional Animal Care and Use Committee of Yeungnam University (AEC2022-022) were employed to perform the work.

#### 4.2.5. Cell Proliferation Assay

MTS assay used to compare the proliferation rates of cells treated with LXG or 5HLXG for 4 days after treatment. Cells treated with LXG or 5HLXG at different concentrations were incubated in CellTiter 96^®^ AQueous One Solution Reagent (Promega, Madison, WI, USA) for 1 h in a humidified 5% CO2 incubator at 37 °C, and absorbances were measured at 490 nm using a microplate reader (Biotek Synergy H1, Winooski, VT, USA).

#### 4.2.6. Immunocytochemistry

Immunocytochemistry was performed using MYH anti-body. C2C12 cells were washed with PBS, fixed with 4% formaldehyde, and permeabilized with 0.2% Triton X-100. Cells were incubated overnight with MYH antibody, followed by Alexa Fluor 488 secondary antibody. After washing, nuclei were counter-stained with DAPI, and fluorescence imaging was conducted using a fluorescence microscope.

#### 4.2.7. Creatine Kinase Activity Test

Creatine kinase activity in cell lysates was measured using the EnzyChrom™ Creatine Kinase Assay Kit (BioAssay Systems, Hayward, CA, USA). Briefly, 10 μL of lysates were incubated with substrate solution, assay buffer, and enzyme mix. Absorbance was measured at 340 nm using a microplate reader. Activity was calculated using [(OD_25min_ − OD_20min_/OD_CALIBRATOR_ − OD_H2O_) × 600].

#### 4.2.8. Real-Time RT-PCR

Total RNA was isolated from cells using Trizol^®^ reagent (Invitrogen, Waltham, MA, USA), and real-time RT-PCR was conducted as we previously described [27]. Relative gene expression was calculated to non-treated controls and calculated using the 2^−∆Ct^ method, where ∆Ct = Ct gene − Ct control. Glyceraldehyde 3-phosphate dehydrogenase (GAPDH) served as the internal control. PCR primer details are provided in Table S1.

#### 4.2.9. Western Blot Analysis

The MSCs and C2C12 cells were lysed using RIPA buffer (Thermo Fisher Scientific, Waltham, MA, USA). Proteins (50 μg) were separated by 10% SDS-PAGE. Separated proteins were transferred by a PVDF membrane and probed using target protein-specific primary antibodies. Membranes were washed and incubated with secondary antibodies (horseradish peroxidase-conjugated) (goat anti-mouse or anti-rabbit; GeneTex, Irvine, CA, USA). Then, blots analysis was performed using the Dyne ECL Pico Plus Western blotting Detection Kit (Dyne Bio, Seongnam, Republic of Korea). Band images were analyzed by using chemiluminescent imager.

#### 4.2.10. Reactive Oxygen Species (ROS) Levels

The media from cells were removed, treated with 10 µM 2′,7′-dichlorofluorescein, incubated for 2 h at 37 °C, then washed with PBS, and fluorescence measured using a microplate reader.

#### 4.2.11. Statistical Analysis

It is performed by One-way ANOVA in SAS ver. 9.0 (SAS Institute, Cary, NC, USA). The *p* values < 0.05 were considered for statistical significance.

## 5. Conclusions

**Figure 7:**
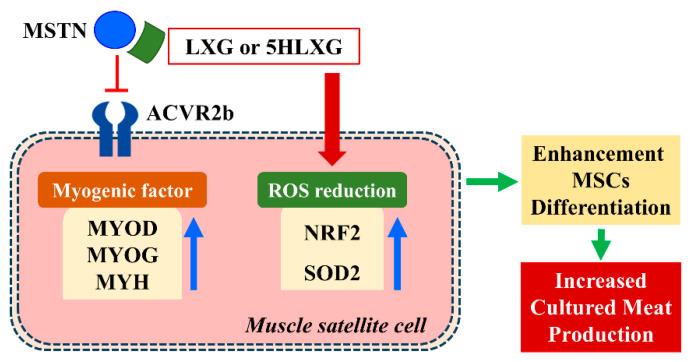
Inhibition of MSTN for SM development during CM production. LAX or 5HLAX inhibited MSTN and ROS production, increased the expressions of muscle regulatory factors, and thus, enhanced myogenesis. LAX and 5HLAX both increased NRF2 and SOD2 expressions and downregulated ROS levels.

CM production is currently being adopted for the future supply of proteinaceous foods, and novel techniques are required to improve many aspects of the production process. In the present study, LAX and 5HLAX were used as supplements in MSC culture media to test their effects on the proliferation and differentiation of MSCs. Both compounds were found to significantly enhance differentiation at 10 nM and reduce ROS production in media. In addition, LAX and 5HLAX supplementation supported myotube formation and maturation, which are important for large-scale CM production. Hence, the study indicates that LAX and 5HLAX have potential use as media supplements and benefit CM production. A putative mechanism underlying the activities of LXG and 5HLXG is provided in Figure 7.

## Figures and Tables

**Table 2:** The properties of LXG and 5HLXG.

Parameters		LXG	5HLXG
Molecular Formula		C_27_H_42_O_4_	C_27_H_42_O_5_
Molecular Weight (g/mol)		430.6	446.6
Absorption	human intestinal absorption	97.12%	96.78%
Toxicity	AMES toxicity	No	No
	Hepatotoxicity	No	No
	Skin Sensitization	No	No

## Data Availability

Data will be made available on request.

## Supplementary Materials

The following supporting information can be downloaded at: https://www.mdpi.com/article/10.3390/ijms26010345/s1.
