# Supplementary material for: The Effects of Laxogenin and 5-Alpha-hydroxy-laxogenin on Myotube Formation and Maturation During Cultured Meat Production
Source: Int J Mol Sci. 2025 Jan 2;26(1):345. doi: 10.3390/ijms26010345 (PMC11720223; doi:10.3390/ijms26010345)

Supplementary table 1.

Table S1. Primer information

Supplementary Table 1.

	Gene	Species	Product size (bp)	Tm (°C)	Forward primer	Reverse primer
1	GAPDH	Mouse	155	59	5′- TGC TGG TGC TGA GTA TGT CG- 3′	5′- CAA GCA GTT GGT GGTACA GG - 3′
2	MYOD	Mouse	213	59	5′- AGG AGC ACG CAC ACT TCT CT - 3′	5′- TCT CGA AGG CCT CAT TCA CT- 3′
3	MYOG	Mouse	185	59	5′- TCC AGT ACA TTG AGC GCC TA- 3′	5′- CAA ATG ATC TCC TGG GTT GG- 3′
4	MYH	Mouse	141	59	5′- CTG AAG CAG AGG CAA GTA GTG- 3′	5′- CGA AAT GAG GAT GGG TGC TC- 3′
5	MSTN	Mouse	163	59	5′- ACG CTA CCA CGG AAA CAA TC - 3′	5′- GGA GTC TTG ACG GGT CTG AG - 3′
6	SMAD2	Mouse	203	58	5′- GAG CTC AAG GCA ATC GAA AA - 3′	5′- CCT GCT GGG AAA TTT GTG TT - 3′
7	SMAD3	Mouse	197	59	5′- CTG GGC CTA CTG TCC AAT GT - 3′	5′- GGT GGG ATC TTG CAG ACA GT - 3′
8	ACVR2b	Mouse	197	58	5′- AAC TTC CAG AGA GAC GCC TT - 3′	5′- ATC GTG GGC CTC ATC TTC TT - 3′
9	NRF2	Mouse	100	59	5′- AAC AGA ACG GCC CTAAAG CA - 3′	5′- TGG GATTCA CGC ATAGGA GC - 3′
10	SOD2	Mouse	170	59	5′- AAC GCC ACC GAG GAG AAG TA - 3′	5′- TCC AGC AAC TCT CCT TTG GGT - 3′
11	GAPDH	Bovine	208	59	5′- GGG TCA TCT CTG CAC CT - 3′	5′- ACA GTC TTC TGG GTG GCA GT - 3′
12	MYOD	Bovine	229	59	5′- GAT GAC CCG TGT TTC GAC TC - 3′	5′- TAG TCG TCT TGC GTT TGC AC - 3′
13	MYOG	Bovine	197	59	5′- TGG GCG TGT AAG GTG TGT AA - 3′	5′- TGC AGG CGC TCT ATG TAC TG - 3′
14	MYH	Bovine	235	57	5′- GGA GAT GCG AGA TGA AAA GC - 3′	5′- CAT GTT GGC CAT TTC CTT CT- 3′
15	MSTN	Bovine	240	59	5′- TGC CCA CGG AGT CTG ATC TT - 3′	5′- CAG TGC CTG GGT TCA TGT CA - 3′
16	GAPDH	Porcine	147	60	5′- TCG GAG TGA ACG GAT TTG GC - 3′	5′- TGC CGT GGG TGG AAT CAT AC - 3′
17	MYOD	Porcine	196	60	5′- GCA CTA CAG CGG TGA CTC AG - 3′	5′- CAC GAT GCT GGA CAG ACA GT - 3′
18	MYOG	Porcine	164	58	5′- CAG TGA ATG CAG TTC CCA CA - 3′	5′- CCA CAT CCT CCA CTG TGA TG - 3′
19	MYH	Porcine	162	58	5′- TTC CTT CCA AAC CGT CTC TG - 3′	5′- TTA CAC CTC AGC TGG TGC AG - 3′
20	MSTN	Porcine		59	5′- CAT GCC TAC AGA GTC TGA TCT T - 3′	5′- CAG TGC CTG GGT TCA TGT CA - 3′
21	GAPDH	Chicken	157	59	5′- CAA CAT CAA ATG GGC AGA TG- 3′	5′- GAC ACC CCA TCA CAA ACA TGG- 3′
22	MYOD	Chicken	160	59	5′- AGC TCT CGC AGG AGA AAC AG- 3′	5′- CTG GAG GCA GTA TGG GAC AT- 3′
23	MYOG	Chicken	205	59	5′- CGC CAT CAG TAC AAT CGA G- 3′	5′- ATC GCT CAG GAG GTG ATC TG- 3′

Supplementary figures

Figure S1. Proliferation of bovine MSCs treated with different concentrations of LXG or 5HLXG. A & C) Cell morphologies observed under a microscope. B & D) Cell proliferations were evaluated using an MTS assay.

Figure S1.

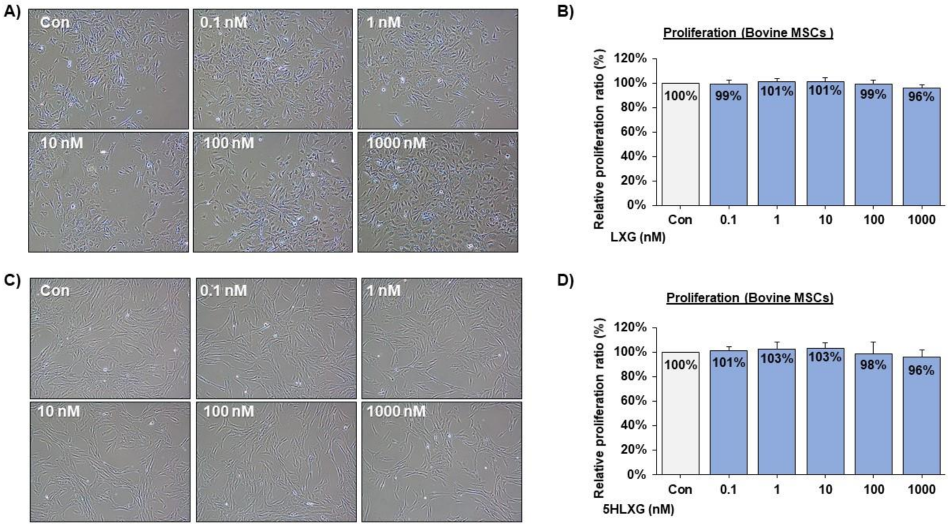

Figure S2. Proliferation of porcine MSCs treated with different concentrations of LXG or 5HLXG. **A & C)** Cell morphologies observed under a microscope. **B & D)** Proliferations were evaluated using an MTS assay**.**

Figure S2.

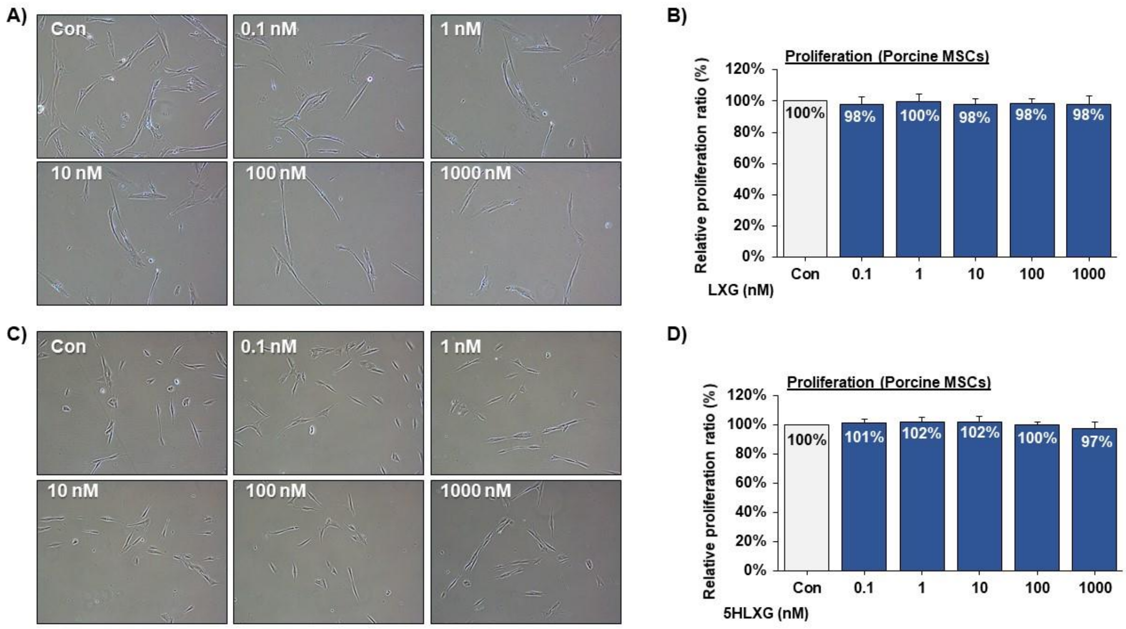

Figure S3. Proliferation of chicken MSCs treated with different concentrations of LXG or 5HLXG. **A & C)** Cell morphologies observed under a microscope. **B & D)** Proliferations were evaluated using an MTS assay**.**

Figure S3.

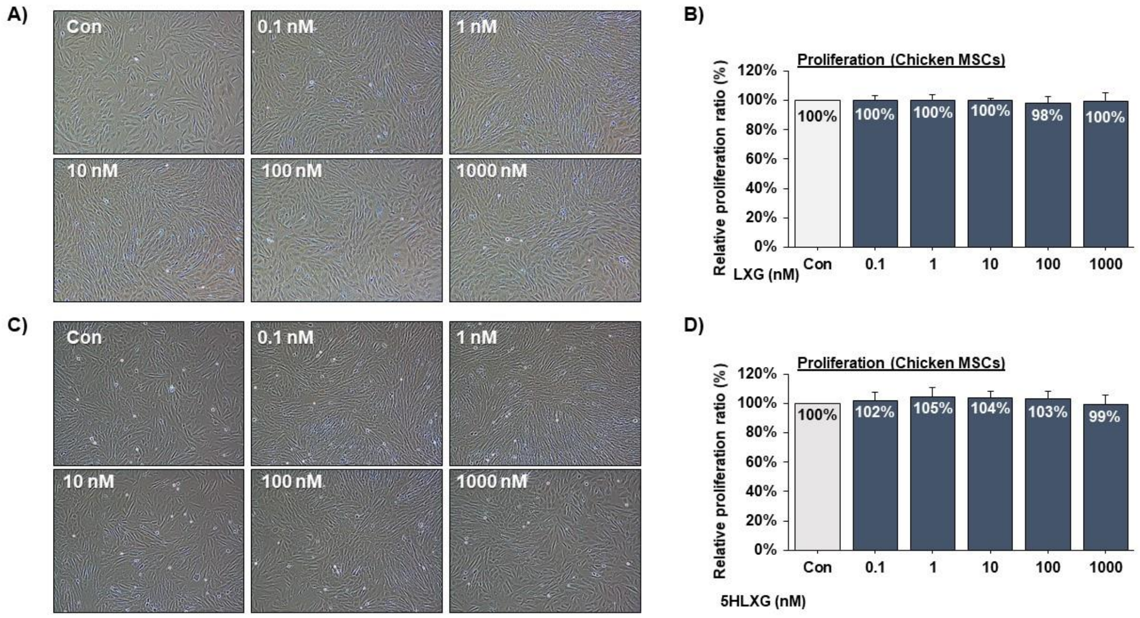

Figure S4. Proliferation of C2C12 cells treated with different concentrations of LXG or 5HLXG. **A & C)** Cell morphologies observed under a microscope. **B & D)** Proliferations were evaluated using an MTS assay**.**

Figure S4.

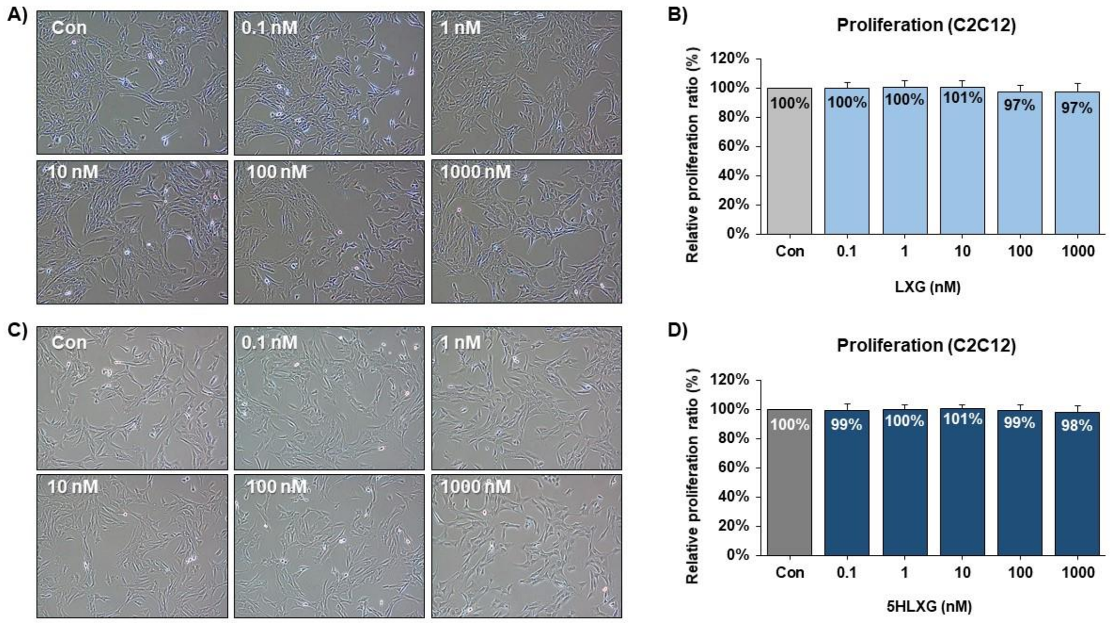

Figure S5. Differentiation of bovine MSCs treated with different concentrations of LXG or 5HLXG. **A & B)** Cell morphologies observed under a microscope.

Figure S5.

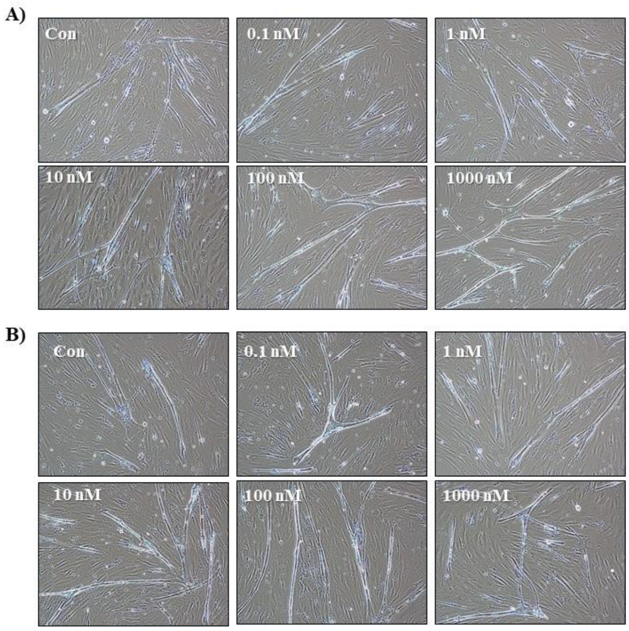

Figure S6. Differentiation of porcine MSCs treated with different concentrations of LXG or 5HLXG. **A & B)** Cell morphologies observed under a microscope.

Figure S6.

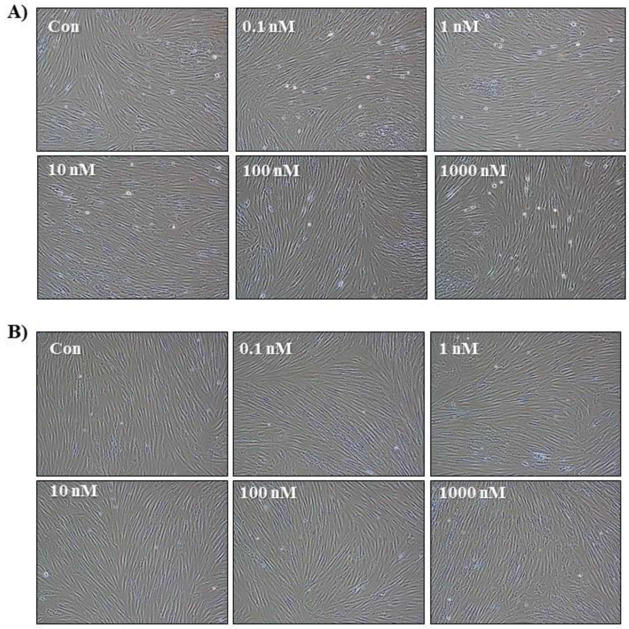

Figure S7. Differentiation of chicken MSCs treated with different concentrations of LXG or 5HLXG. **A & B)** Cell morphologies observed under a microscope.

Figure S7.

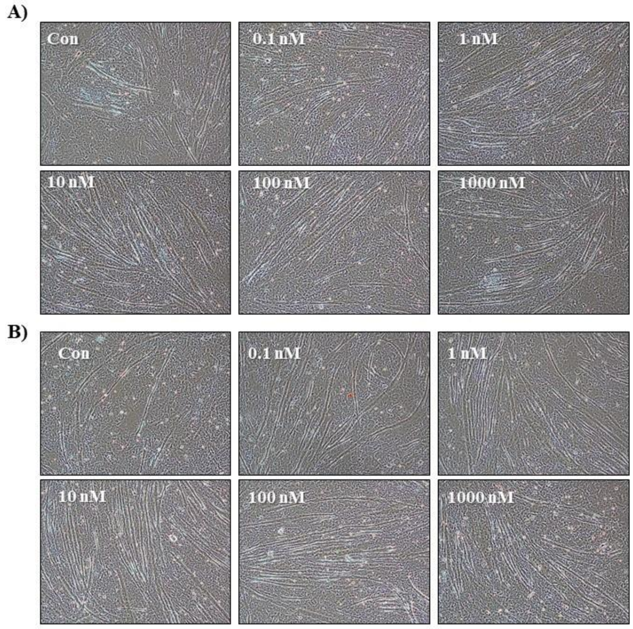

Figure S8. Differentiation of C2C12 cells treated with different concentrations of LXG or 5HLXG. **A & B)** Cell morphologies observed under a microscope.

Figure S8.

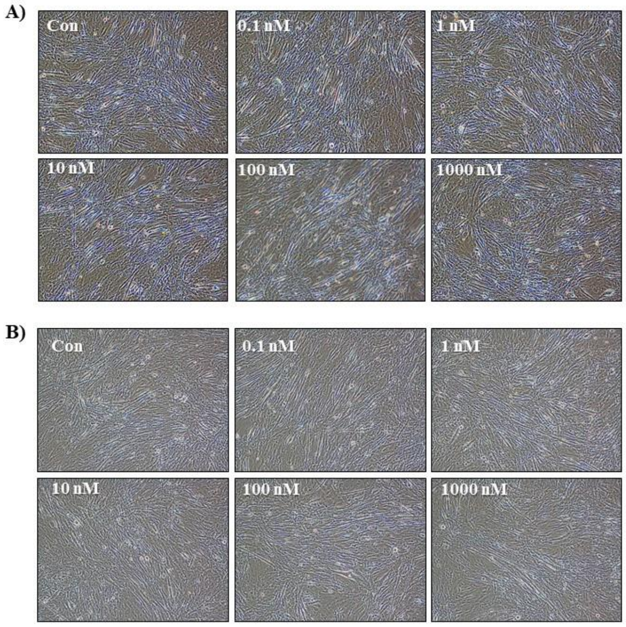

Supplement: Supplementary file 1 [file ijms-26-00345-s001.zip › ijms-3380446-supplementary.pdf]
